# From Follicle Development to Fertilization: How PCBs and PFAS May Shape Female Reproductive Health

**DOI:** 10.3390/toxics14090780

**Published:** 2026-09-03

**Authors:** Md Hasanur Alam, Elise C. Barteld, Julia Tlapa, Monica Ridlon, Kimberly P. Keil Stietz

**Affiliations:** Department of Comparative Biosciences, School of Veterinary Medicine, University of Wisconsin-Madison, Madison, WI 53706, USAkkeil@wisc.edu (K.P.K.S.)

**Keywords:** fertilization, folliculogenesis, PCBs, PFAS, reproductive toxicity

## Abstract

Endocrine system hormones regulate growth, metabolism, and reproduction. These hormones often function at low concentrations, making them especially susceptible to disruption via endocrine-disrupting chemicals (EDCs) that can mimic, block, or disturb normal hormone signaling. Among these EDCs, polychlorinated biphenyls (PCBs) and per- and polyfluoroalkyl substances (PFAS) are of special concern for human health because they are persistent and bioaccumulate. In this review, we focus on effects of PCBs and PFAS on female reproduction, with emphasis on steroidogenesis, ovarian function, fertility, and assisted reproductive technology (ART) outcomes. We describe the chemical properties and exposure routes of PCBs and PFAS, and how these features explain their long half-lives and ability to transfer to the female reproductive system and fetus. Evidence suggests that both PCBs and PFAS can reduce follicle numbers, disturb oocyte quality, and increase oxidative stress and apoptosis. Human and animal data also link PCB and PFAS exposure with altered ART outcomes. Together, the literature indicates that persistent exposure to PCBs and PFAS poses a significant and long-lasting risk to female reproductive health.

## 1. Introduction

Endocrine system hormones are crucial regulators of growth, development, metabolism, and reproduction. Their biological activity often depends on tightly regulated low concentrations and precise signaling dynamics. Therefore, they are highly susceptible to exogenous chemicals, especially endocrine-disrupting chemicals (EDCs) that mimic, block, or disturb hormone signaling important for female reproductive regulation.

Compounds or chemicals which interfere with female reproductive function are often referred to as reproductive toxicants. Reproductive toxicants may act not only through direct cytotoxicity, but also through disruption of hormone homeostasis, subtle perturbations of cell signaling, or interference with developmental programming. Among the many toxicants that cause endocrine disruption, PCBs and PFAS are of particular interest due to their widespread exposure, persistence and bioaccumulation, and evidence of reproductive effects [1].

### 1.1. Polychlorinated Biphenyls (PCBs)

**Figure 1:**
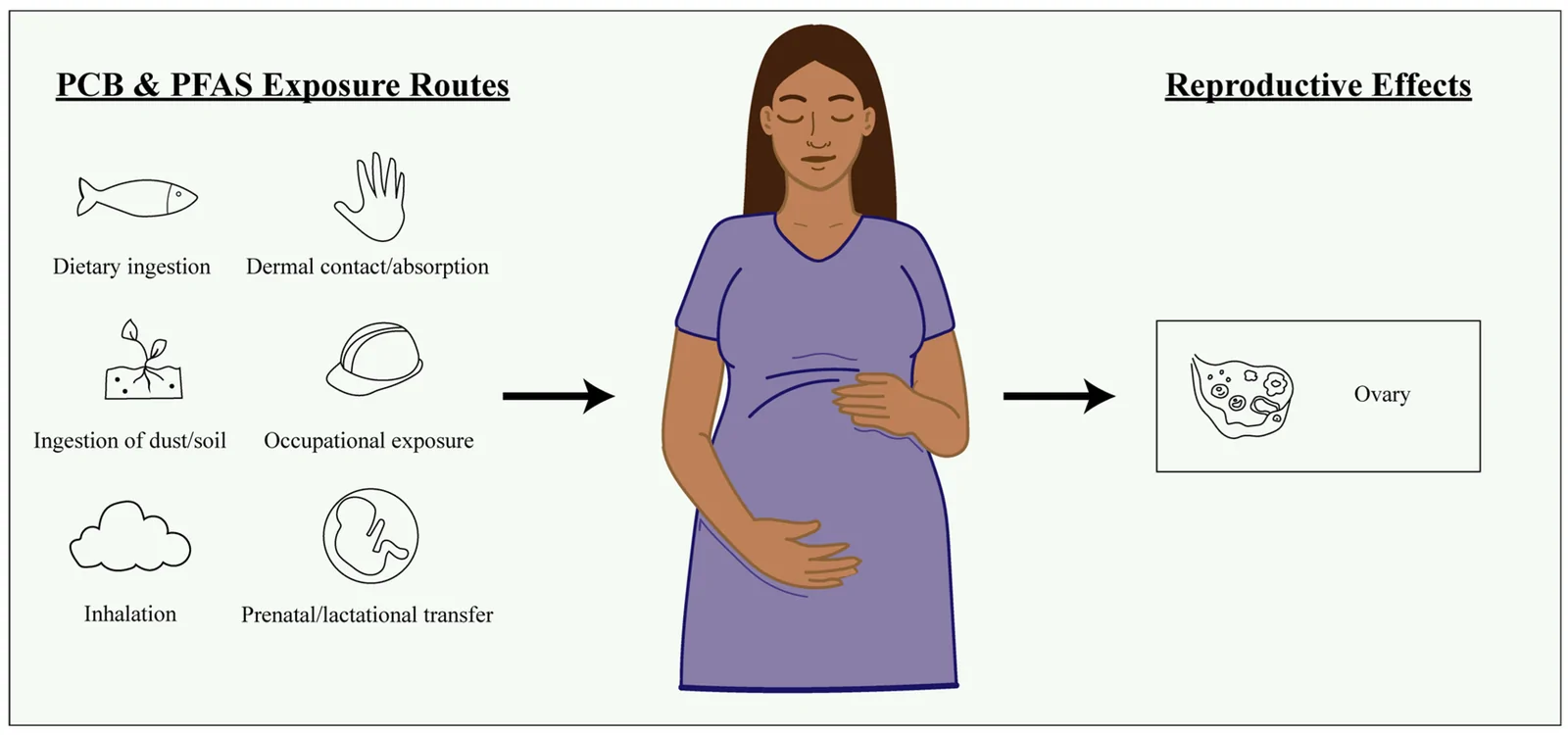
Common routes of exposure to PCBs and PFAS and their effects on reproduction.

PCBs are man-made aromatic chemicals characterized by a biphenyl backbone substituted with varying numbers and positions of chlorine atoms. In total, 209 distinct PCB congeners exist, each differing in molecular structure and biological activity [2]. Depending on the degree and position of ortho-chlorine substitutions, PCBs can have a coplanar structure or a noncoplanar configuration due to steric hindrance between the ortho-substituted chlorine atoms [3]. These structural differences can influence receptor binding, metabolism, and mechanism of action for PCBs. For example, coplanar PCBs are also called dioxin-like PCBs because they can bind the aryl hydrocarbon receptor (AhR) and elicit responses similar to the more potent AhR ligand 2,3,7,8-tetrachlorodibenzo-p-dioxin (TCDD) [3]. Noncoplanar PCBs are often termed non-dioxin-like and do not typically bind AhR but rather act through other mechanisms, including binding of ryanodine receptors and hormone receptors [3,4,5]. PCBs were manufactured and sold globally under several trade names, including Clophen (Germany), Aroclor (USA), Kanechlor and Santothrem (Japan), and Phenoclor and Pyralene (France) [6]. PCBs were once widely used as industrial fluids, insulating materials, and plasticizers [7,8]. PCBs were incorporated into hydraulic and lubricating fluids, inks, adhesives, carbonless copy paper, flame retardants, and construction materials [7]. Due to mounting health and environmental concerns, PCBs were restricted under the Toxic Substances Control Act of 1976 and PCB production was banned in the USA in the late 1970s and restricted or eliminated more globally in the 2000s [5]. However, PCBs continue to persist and accumulate due to their stability, resistance to degradation, and lipophilicity, which promote bioaccumulation and biomagnification [8,9,10,11]. In addition to legacy sources of PCBs that were produced prior to the ban and remain in the environment, contemporary sources of PCBs also contribute to ongoing exposure [5]. Contemporary PCBs are released as unintentional byproducts from industrial processes, for example, in paint pigment production [12,13,14]. In humans, the predominant route of PCB exposure is via consumption of contaminated food, especially lipid-rich products like fish, meat, milk, and eggs [15,16,17,18] (Figure 1). Other routes of exposure include inhalation of PCB-contaminated air or dust, particularly in old buildings [19,20], as well as dermal contact with PCB-contaminated materials, soil, or dust [11]. Early life exposure can also occur through placental and lactational transfer [21].

### 1.2. Polyfluoroalkyl Substances (PFAS)

PFAS are a class of synthetic organofluorine compounds characterized by carbon chains in which hydrogens have been fully or partially replaced by fluorine atoms. More than 4700 PFAS have been identified to date [22]. PFAS are grouped into three broad classes: perfluoroalkyl acids (PFAAs), their precursor compounds, and other PFAS such as fluoropolymers and perfluoropolyethers (PFPEs) [22]. Among these groups, PFAAs are the most extensively studied, with perfluorooctanoic acid (PFOA) and perfluorooctanesulfonic acid (PFOS) being the most frequently detected in environmental samples [23]. PFAS are highly stable and resistant to degradation, and as such, have been widely used in industrial applications, water-repellent and non-stick consumer products, firefighting foams, and food packaging [24,25,26]. PFAS have been detected in food, drinking water, consumer products, indoor dust, and cooking materials, leading to ubiquitous human exposure [23,27]. PFAS are often referred to as “forever chemicals” due to their chemical stability and persistence in the environment. Many PFAS also have long biological half-lives, allowing them to bioaccumulate in organisms over time [28]. Together, their chemical stability, resistance to degradation, and prolonged biological retention can result in cumulative exposure over time. Consequently, even low-level, chronic exposure can likely result in biologically meaningful body burdens with the potential to disrupt reproductive and other systems. While not banned as a whole class, measures are in place in the USA and other countries to limit PFAS exposures. In the USA, the Environmental Protection Agency (EPA) has issued health advisories for PFAS in drinking water and established reporting and testing requirements under the Toxic Substances Control Act [26]. Many individual PFAS compounds are restricted, being phased out or are heavily regulated [25]. However, due to their persistence, these compounds remain a contemporary health concern. The primary route of human exposure is oral exposure [29,30,31,32,33,34,35]. PFAS can also be present in indoor air as volatile precursors or aerosolized particulates from consumer products, leading to inhalation routes of exposure [36,37,38]. Although dermal absorption of PFAS is generally considered limited for many, it can occur [39,40]. Early life PFAS exposure can occur through placental transfer and breastfeeding [41,42,43]. Occupational exposure also exists and is higher in workers in PFAS manufacturing, firefighting, or chemical use (e.g., fluorochemical plants, carpet/fabric production) [44,45].

Figure 1 illustrates major routes through which PCBs and PFAS can enter the human body. Both chemicals share multiple exposure pathways, including dietary ingestion, inhalation, ingestion of dust or soil, dermal contact or absorption, prenatal and lactational transfer, and occupational exposure. These exposures are associated with adverse effects on key reproductive tissues.

The female reproductive system is tightly regulated and includes processes of oogenesis, folliculogenesis, ovulation, hormone homeostasis, fertilization, implantation, and placentation. These processes are highly sensitive to fluctuations in hormone concentrations, temporal regulation, and metabolic crosstalk, making them potentially vulnerable to chemical exposures. Ovaries contain a finite number of follicles at birth, leading to an overall decline in oocyte reserves over time [46]. Chemical exposures may shorten reproductive lifespan by altering ovarian reserve or disturbing hormone signaling. In human and animal studies, PCB exposure has been associated with altered menstrual cyclicity, earlier menopause, endometriosis, ovarian insufficiency, epigenetic changes, and reduced fertility [47,48,49,50,51,52]. Similarly, PFAS exposure has been linked to disruptions in menstrual cycles, altered hormone levels, delayed fecundability, ovarian dysfunction, and pregnancy loss in women [53,54,55,56,57]. In addition, both PCBs and PFAS can transfer to the next generation through the placenta or via lactation, making maternal exposures during pregnancy or lactation particularly concerning [58,59]. PCBs and PFAS act as EDCs through a combination of overlapping and distinct mechanisms, and thus, comparing their effects in an integrated review may reveal common vulnerabilities of female reproduction to persistent environmental pollutants.

## 2. Effects of PCBs and PFAS on Ovarian Steroidogenesis

**Figure 2:**
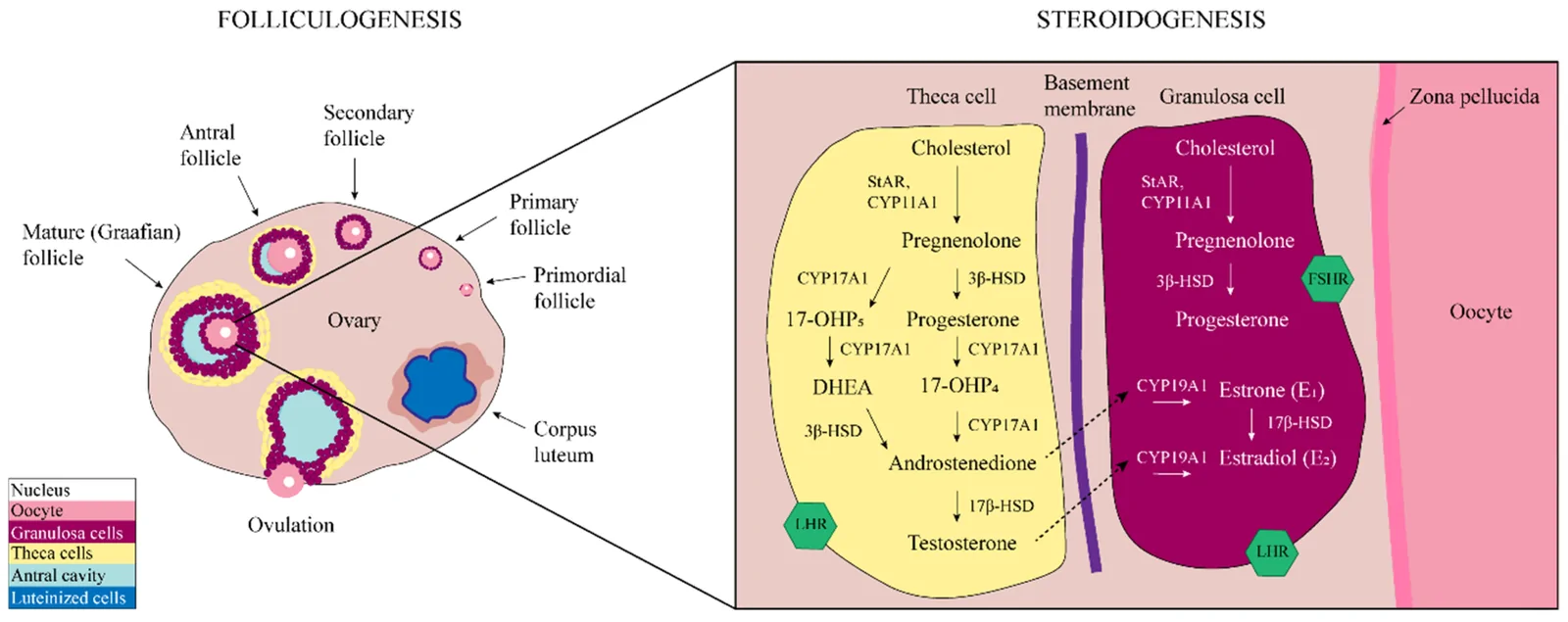
Ovarian follicular structure and estrogen biosynthesis via the two-cell, two-gonadotropin model. Cross-section of the ovary showing major follicular compartments, including the oocyte, granulosa cells, theca cells, antral cavity, and luteinized cells (**left**). In theca cells, LHR-driven steroidogenesis converts cholesterol to androgens (androstenedione and testosterone) through sequential actions of StAR, CYP11A1, CYP17A1, and 3β-HSD (**right**). These androgens are transferred to granulosa cells, where FSHR signaling facilitates their aromatization by CYP19A1 to estrone (E_1_) and estradiol (E_2_). Abbreviations: StAR, steroidogenic acute regulatory protein; CYP11A1, cytochrome P450 11A1; CYP17A1, cytochrome P450 17A1; 3β-HSD, 3β-hydroxysteroid dehydrogenase; CYP19A1, aromatase; 17β-HSD, 17β-hydroxysteroid dehydrogenase; LHR, luteinizing hormone receptor; FSHR, follicle-stimulating hormone receptor; E_1_, estrone; E_2_, estradiol. Solid arrows indicate enzymatic conversion steps; dashed arrow indicates diffusion of androstenedione from the theca cell to the granulosa cell across the basement membrane (**right**).

The ovary contains cortical follicles at sequential developmental stages, from primordial to preovulatory, each composed of an oocyte encased by granulosa cells and surrounded by a theca cell layer (Figure 2). During folliculogenesis, granulosa cells proliferate and form the antrum, while theca cells differentiate and acquire steroidogenic capacity. According to the two-cell, two-gonadotropin model, luteinizing hormone (LH) stimulates theca cells to synthesize androgens from cholesterol, and follicle-stimulating hormone (FSH) induces granulosa cells to convert these androgens into estradiol via aromatase (CYP19A1), a process essential for follicular maturation and ovulatory competence [60]. At various stages, ovarian cells can express distinct hormone receptors; for example, granulosa cells express FSH receptor (FSHR), LH choriogonadotropin receptor (LHCGR), estrogen receptor beta (ERβ), and progesterone receptor (PGR), whereas theca cells primarily express LHCGR to support androgen production. The ovarian surface epithelium expresses multiple steroid and growth factor receptors, including androgen receptor (AR), estrogen receptor alpha (ERα), progesterone receptor (PR), gonadotropin-releasing hormone receptor (GnRH-R), and others, contributing to ovarian growth regulation and endocrine responsiveness [61]. Because steroidogenesis relies on tightly regulated granulosa-theca interactions, it is particularly vulnerable to disruption by endocrine-disrupting chemicals such as PCBs and PFAS.

Measures of steroidogenesis include all reproductive steroid hormones and their intermediates, as well as the pituitary hormones which may influence the stimulation of steroid hormones [62,63,64,65]. PCB exposure has been shown to disrupt steroidogenesis in animal in vitro and in vivo models. Addition of PCB 126, PCB 77, PCB 153, and Aroclor 1248 to bovine luteal cells in culture has been shown to increase oxytocin (OT) secretion, with the response varying among PCB congener, follicle size and estrous stage [64,66]. When these PCBs were administered in the presence of LH, their effects on OT secretion were mixed, either increased or decreased depending on the PCB congener and estrous stage of the luteal cells [66]. In contrast, PCB 126, PCB 77 and PCB 153 decreased LH-stimulated progesterone (P_4_) secretion by bovine luteal cells [66]. Given that these effects were observed in the presence of dioxin-like and estrogenic PCB congeners, these findings suggest involvement of ER- and AhR-receptor-mediated pathways [64,66]. These findings suggest that PCBs may disrupt luteal function by inhibiting LH-stimulated steroidogenesis (P_4_) while altering OT secretion from the CL in a congener- and estrous stage-dependent manner. PCB 153 and PCB 126 have also been shown to disrupt steroidogenesis in porcine granulosa and theca cells by altering the conversion of steroid precursors. Both PCBs have been shown to inhibit cholesterol mobilization, which consequently affects hormone synthesis, including pathways involved in T and E_2_ secretion potentially via effects on 17β-hydroxysteroid dehydrogenases and P450 aromatase activity, respectively [62]. In addition to alterations in hormone production, evidence also suggests that PCBs can influence receptor abundance. PCB 126 administered to Sprague–Dawley rat dams through gestation and lactation (3 µg/kg/day) resulted in decreased expression of FSH and LH receptors in offspring ovaries at post-natal day 15 and 24 respectively [67].

PFAS exposure has also been shown to disrupt steroidogenesis in in vitro and in vivo models. PFAS compounds can disrupt normal granulosa cell function [68]. When exposed specifically to the PFAS chemical PFOA, HGrC1 cells (human nonluteinized granulosa cell line) show increased proliferation and altered expression of cell cycle genes and those involved in the Hippo pathway [69]. In vitro, a mixture of PFOA, PFOS, and perfluorohexanesulfonic acid (PFHxS) induced secretion of P_4_ under basal and FSH-stimulated conditions, upregulated steroidogenic acute regulatory (StAR) protein (crucial for steroid hormone biosynthesis) and induced transcriptomic changes in human granulosa cells [68]. Porcine granulosa cells exposed to PFOA in vitro are shown to have lower cell proliferation rates, altered redox homeostasis, and disrupted steroidogenesis, exhibiting increases in 17β-estradiol at all doses and having both stimulatory and inhibitory effects on P_4_ at low or high concentrations respectively [70,71]. A study using a PFAS mixture in mouse granulosa cells found decreased expression of the steroidogenic enzyme 3-beta-hydroxysteroid dehydrogenase and decreased production of P_4_ and E_2_; the PFAS mixture also led to reduced mitochondrial function and an increase in reactive oxygen species (ROS) and lipid peroxidation [72]. C8-C14 perfluorocarboxylic acids (PFCAs) and C8S polyfluorosulfonic acids (PFSAs) have also been shown to inhibit the function of 17-beta-hydroxysteroid dehydrogenase isoform 1 (17β-HSD1), an estrogen-metabolizing enzyme necessary for steroidogenesis in the rat ovary, by binding the NADPH and steroid binding site of 17β-HSD1 [73]. Overall, these variable and sometimes opposing findings likely reflect differences in PFAS compound and mixture composition, concentration used, and experimental model (human versus porcine versus mouse granulosa cells), highlighting the context-specific effects of PFAS on steroidogenesis and suggesting that individual PFAS congeners and their mixtures may differentially affect steroidogenic enzymes, hormone production, and mitochondrial function across species and exposure paradigms.

### Effects on Estrogen, Progesterone and Testosterone

PCBs and PFAS have also been shown to alter levels of estrogen in preclinical in vitro and in vivo models. PCB 153 (non-dioxin-like PCB) decreased E_2_ and increased P_4_ levels in the media of cultured porcine granulosa cells; however, PCB 126 (dioxin-like PCB) led to an increase in E_2_ and P_4_ but only at the highest concentration [74]. These results illustrate the congener-specific effects of PCBs on hormone-related endpoints. PCB 153 was also found to lower E_2_ and raise P_4_ in pig small antral follicle (1–3 mm in diameter) cells in vitro [75]. Culture of porcine small, medium, and large preovulatory follicles with addition of PCB 126 or PCB 153 has been shown to disrupt estradiol secretion, acting to both stimulate and suppress levels dependent upon stage of follicle development and time of exposure [76]. In contrast to in vitro applications of PCB 126, PCB 126 administered to Sprague–Dawley rat dams at 13–19 days postconception reduced E_2_ and P_4_ serum levels in 50-day-old offspring [77]. Female rats given PCB 126 at four weeks of age had reduced serum estradiol and progesterone levels four weeks later; this effect was dependent upon aryl hydrocarbon signaling (the target of dioxin-like PCBs like PCB 126) as rats lacking AhR were protected from this PCB effect [78]. Together, these results demonstrate that PCBs can alter steroids in porcine in vitro culture models and influence circulating steroid levels in juvenile or developmentally exposed rats, further illustrating the importance of timing and congener concentrations in eliciting alterations related to steroid hormone secretion.

PFAS are also known to disrupt estrogen levels and signaling pathways. Oral administration of perfluorododecanoic acid (PFDoA) to prepubertal female rats for 28 days diminished estradiol levels in the ovary and altered gene expression of enzymes responsible for estrogen synthesis and cholesterol transport in a dose-specific manner [79]. They also observed a reduction in estrogen receptor alpha (ERα) and estrogen receptor beta (ERβ) mRNA expression in the rat ovary [79]. PFOS, PFOA, and fluorotelomer alcohol (FTOH) have also been shown to have estrogenic activity potentially mediated by estrogen receptors in fish hepatocyte assays [80]. Similarly, both human and rat ERs were activated by PFHxS, PFOSA, and FTOH in an in vitro assay system [81]. In that study, PFAS was also shown to activate peroxisome proliferator-activated receptors (PPARα and PPARγ), which establishes PPAR binding as a shared, receptor-mediated molecular target for this chemical class [81]. PPARγ activation has been linked to inhibition of aromatase (CYP19A1) in granulosa cells, which may reduce estrogen production. Chronic exposure for four months to a low dose of PFOS (0.1 mg/kg/day) in adult female mice reduced serum estrogen and progesterone levels in mice in proestrus and diestrus, and reduced LH, FSH, and gonadotrophin-releasing hormone in mice in proestrus [82]. *StAR* mRNA expression also decreased in ovaries of PFOS-exposed mice, with evidence suggesting a mechanism involving a reduction in histone H3K14 acetylation of the *StAR* promoter; thus, proposing a mechanism whereby PFOS effects on histone acetylation of *StAR* may regulate E_2_ and contribute to abnormal ovarian function [82].

PCBs and PFAS have also been shown to alter levels of testosterone in preclinical in vitro and in vivo models. PCB 153 and PCB 126 reduced testosterone (T) secretion in theca cells of porcine large follicles over 48 h of culture [62,75,83], and these PCBs were also found to decrease P_4_ in cultured porcine luteal cells over 48 h in culture; however, after 72 h in culture, PCB 153 exposure increased P_4_ secretion [84]. PCB 126 and PCB 153 effects on reduced testosterone and progesterone secretion in culture were reversed in the presence of androstenedione and 20-hydroxylated cholesterol respectively [62]. These results suggest that in co-culture of porcine theca and granulosa cells, PCB 126 and PCB 153 effects are likely due to inhibition of cholesterol mobilization, causing reduced availability of substrate for hormone synthesis in a dose- and timing-specific manner [62]. The effects of PCBs on steroid hormone secretion from porcine theca and granulosa cell cultures may be further complicated by exposure to other PCBs and persistent organic chemicals. A study that used a mixture of PCBs and other non-dioxin-like persistent organic pollutants found that while administration of PCB 126 and PCB 138 alone decreased testosterone secretion, when these PCBs were combined with PCB 153, PCB 118, and PCB 180, there was no effect on testosterone secretion [85]. Similarly, they found that while PCB 153, PCB 118 or PCB 180 increased estradiol secretion alone, this effect was blocked or led to a reduction in estradiol secretion when combined with different combinations of PCB 126 and PCB 138 [85]. Together, this work highlights that PCB effects are dependent upon the mixture composition and this is an important aspect to consider in translational applications of in vitro and preclinical studies [85,86]. PCBs can interact with the androgen receptor (AR) by directly binding the AR’s ligand-binding domain and acting as antagonists [87,88]. Further hydroxylated PCB metabolites, 4,4′-PCB-3-OH and 2′,5′-PCB-3-OH, have been shown to bind to the androgen receptors [89].

PFAS have also been shown to alter secretion of testosterone or reduce substrates such as androstenedione for testosterone production. In a study using porcine thecal cells in culture, addition of low uM concentrations of PFOS led to a reduction in the basal secretion of androstenedione, which was also observed when cells were stimulated with LH [90]. Adult female mice administered PFOA for ten days showed increases in testosterone and *Cyp19a1* levels (only at low 1 mg/kg but not higher concentrations), suggesting disruption of hormone balance and further highlighting the complex and non-linear effects of these compounds on steroidogenesis pathways in the ovary [91]. In an adolescent exposure model, female adolescent mice were exposed to a mixture of PFAS for 15 days, then allowed to recover for an additional 45 days; this adolescent exposure was sufficient to induce increases in ovary expression of steroidogenesis enzymes including *Cyp19a1* as well as other enzymes particularly important for androgen production, *Hsd3b1*, *Cyp17a1*, and *Hsd17b1* [92]. PFAS can also bind and inhibit the AR, reduce AR function and androgen-responsive gene expression, lower AR protein levels, and lead to antiandrogenic effects [93,94]. Three PFAS compounds, namely 9-(nonafluorobutyl)−2,3,6,7-tetrahydro-1 H,5 H,11 H-pyrano [2,3-f]pyrido [3,2,1-ij]quinolin-11-one (NON), 2-(heptafluoropropyl)− 3 phenylquinoxaline (HEP), and 2,2,3,3,4,4,5,5,5-nonafluoro-N-(4-nitrophenyl) pentanamide (NNN), can competitively bind to the AR thus preventing endogenous androgens like testosterone from activating the receptor in human cell lines [93]. In the same studies, PFAS was also shown to decrease expression of downstream AR-responsive genes such as PSA and FKBP5, and in some cases increased AR mRNA itself, indicating that the normal downstream signaling cascade of AR is disrupted by PFAS [93].

Comparing the studies described above, PCB and PFAS effects rely on several common downstream processes that include impaired cholesterol mobilization, altered 17β-HSD activity, and AR antagonism, indicating overlapping targets in the steroidogenic pathway [62,82,87,93]. However, the upstream mechanisms likely differ. PCB, especially the dioxin-like congeners, have AhR-dependent effects [78], while PFAS have been linked to PPAR signaling, oxidative stress, mitochondrial dysfunction, and epigenetic alterations including reduced StAR promoter acetylation [72,81,82]. The variable and sometimes opposing findings, such as the effects of PCB 126 on estradiol [74,76] or the non-monotonic dose–response of PFOA on testosterone [91], reflect the complexity of these chemicals and pathways. These effects are likely dependent on the specific congener or mixture, concentration/dose, timing of exposure, and context such as age, estrous stage or follicle size. Collectively, these findings underscore the importance of standardized dosing approaches and consideration of species-, tissue-, and stage-specific differences when evaluating shared mechanisms of endocrine disruption among PCBs and PFAS. Future studies that directly compare PCBs and PFAS in a single study to evaluate common endpoints are needed to better define shared molecular pathways and determine how these chemicals contribute to adverse effects on ovarian health and function.

## 3. Effects of PCBs and PFAS on Ovarian Function

### 3.1. Folliculogenesis

Folliculogenesis studies have been conducted using a plethora of animal models–rodents, bovine, porcine, and non-human primates (NHPs). Evidence suggests that PCBs can negatively affect ovarian folliculogenesis by decreasing follicle numbers, disrupting hormone secretion, and altering follicular development pathways. This leads to a reduction in the primordial follicle pool, impairing the growth and maturation of follicles, and inducing atresia. Rodent models of PCB exposure have been utilized to investigate the toxicants’ effects on folliculogenesis. Early studies on the effects of PCB 77 show that a single developmental exposure decreased the number of oocytes and ovarian follicles in offspring 28 days after birth; however, multiple doses throughout gestation and lactation led to an increase in germ cell numbers that was dependent upon mouse strain [95,96]. This indicates that timing of exposure and dosing paradigm can determine the direction of the follicular response. Subsequent studies support the finding that timing of exposure is critical to follicle health. Rats prenatally exposed to PCB 126 had significantly fewer antral follicles at 30 and 50 days old, as well as an increased number of degenerate follicles [77]. Sprague–Dawley rats developmentally exposed to Aroclor 1221 from gestation day 8–18 at 1 mg/kg/day showed a reduced number of primordial follicles and total number of follicles at postnatal day 32; this effect was not observed in younger or older animals [97]. Long-Evans rats developmentally exposed to Aroclor 1016 from gestational days 7–13 at 2.5 mg/kg/day had a reduced number of small preantral follicles, total antral follicles, and increased atretic follicles at postnatal day 24; this effect was partially ameliorated in dams given a levo-thyroxine supplement, suggesting thyroid signaling plays a role in PCB effects on small antral follicles [98]. Female rats exposed to Aroclor 1221 for two weeks during adolescence were found to have accelerated folliculogenesis and increased apoptosis in the ovary [99]. A single exposure to PCB 126 during adolescence led to a reduction in the number of secondary follicles, antral follicles and corpora lutea at 8 weeks of age; furthermore, these effects were shown to be mediated via AhR, as AhR null rats were protected from PCB 126-induced changes in follicle counts [78]. Taken together, these studies clearly show that prenatal exposure to PCBs negatively impacts folliculogenesis and importantly highlight that the timing of exposure as well as concentration are likely to play key roles in determining effects on follicle number and health.

PFAS exposure has been shown to disrupt folliculogenesis by altering oocyte development [100,101,102,103]. In female rats, exposure of 0.1 and 1 mg PFOA/kg/day or 0.1 and 10 mg PFOS/kg/day during postnatal day 1–5 reduced the number of ovarian primordial follicles, growing follicles and corpora lutea in young adults [104]. Interestingly, this effect on follicle number was not observed when animals were administered PFOA or PFOS from postnatal day 26–30, again demonstrating the importance of timing of exposures during key developmental windows [104]. Adolescent exposure to a PFAS mixture for 15 days led to a reduction in primordial and primary follicles in adults at postnatal day 90 [92]. PFOS at a low dose of 0.1 mg/kg/day for four months in adult female mice led to a decrease in the numbers of mature follicles and corpora luteum, with increased follicle atresia [82]. Similarly, adult female mice administered PFOA from gestational days 1–7 or 13 showed reduced numbers and sizes of corpora lutea accompanied by decreased mRNA expression of *StAR*, *Cyp11a1* and *Hsd3b1* in the maternal ovaries [105]. Pregnant mice administered perfluorobutanesulfonic acid (PFBS) at 200 and 500 mg/kg/day on day 1–20 of gestation result in female offspring with reduced ovarian size and weight, and number of ovarian follicles (primordial, primary, secondary, and antral), delayed onset of oestrus, prolonged diestrus and disrupted thyroid hormone synthesis [106]. Others have also reported that thyroid hormones decrease with increased PFAS concentrations [107,108,109].

The studies outlined above indicate that both PCBs and PFAS can disrupt folliculogenesis in a dose- and exposure-window-specific manner, with effects varying across the developmental and adolescent period and also sometimes showing opposite effects [95,96,104,106]. There are likely unique and conserved mechanisms underlying these effects. For example, an AhR-mediated mechanism for dioxin-like PCB 126 has been reported [78], whereas thyroid hormone signaling may represent a shared pathway through which both PCBs and PFAS affect follicular development. Since thyroid hormones play an essential role in ovarian follicular development [110,111,112,113], and this pathway can be altered by PCBs and PFAS [98,106], this pathway represents a plausible shared mechanism through which these environmental contaminants may affect ovarian function and warrants further study.

### 3.2. Oocyte Maturation and Meiotic Defects

The effects of PCBs and PFAS on reproductive abnormalities are summarized in Table 1. PCBs, at a range of concentrations in experimental models, have been shown to disrupt mammalian oocyte maturation by interfering with cellular communication, spindle assembly, cortical granule function, and maternal mRNA regulation [114,115]. Aroclor 1254 exposure to mouse immature oocytes in vitro induced abnormal spindle formation, DNA damage and apoptosis in cumulus cells [116,117]. Aroclor 1254 exposure to bovine oocytes in vitro decreased the number of cells reaching metaphase II and impaired fertilization and maturation [114]. In vitro studies using bovine oocytes exposed to PCB 153 or PCB 126 found that PCB 153 only reduced the percentage of cleaved oocytes, whereas PCB 126 reduced oocyte maturation and blastocyst development in dose-specific manners [118]. This study also highlights the importance of congener-specific effects of PCBs on oocyte maturation. Aroclor 1254 exposure to porcine oocytes decreased the number of zygotes that developed to the blastocyst stage, altered microtubules, and perturbed gap-junction-mediated signaling to cumulus cells during in vitro maturation [119]. In addition, PCBs have also been shown to induce apoptosis. In cultures of bovine cumulus–oocytes, Aroclor 1254 exposure induced apoptosis in cumulus cells [120]. Further in this same culture system, it was demonstrated that PCB mixtures induced cumulus cell apoptosis in a congener-specific manner, with dioxin-like PCBs (PCB 77, PCB 126 and PCB 169) reducing maturation and inducing apoptosis while non-dioxin-like PCBs (PCB 52, PCB 101 and PCB 153) did not [120]. PCB 126 has also been shown to induce apoptosis in granulosa cells of cultured mouse ovaries along with increased expression of apoptotic pathway genes [121]. In the Chinese Hamster Ovary (CHO-K1) cell line, exposure to PCB 77 and PCB 153 can also induce cellular apoptosis [122,123]. This model system also demonstrates that the type and degree of toxicity of PCBs on the ovary is likely linked to the specific structure of the individual congener; toxic effects of PCB 153 on CHO-K1 cells in vitro were mitigated by exposure to the antioxidant resveratrol; however, the toxic effects of PCB 77 were not [122]. Taken together, these studies illustrate that PCBs can impair oocyte quality, decrease fertilization rates, and lower reproductive success. Further, there are likely dose- and cell type-specific effects, with evidence that PCB structure may influence toxicity as well as the response to therapeutic intervention strategies.

PFAS exposure adversely affects oocyte maturation and meiosis. PFAS exposure can lead to mitochondrial dysfunction, oxidative stress, and meiotic spindle assembly defects, leading to oocyte maturation failure [128,129,130]. Studies have also shown that PFAS disrupts cytoskeletal organization and induces apoptosis in oocytes [128]. To illustrate the mechanism, exposure to a high concentration (350–450 μM) of perfluorodecanoic acid (PFDA), a member of the PFAS family, was shown to cause deficits in meiosis and cytoskeleton arrangement, mitochondrial dysfunction and elevated levels of reactive oxygen species and apoptosis in cultured mouse oocytes [128]. Similarly, exposure to PFOS in porcine oocytes in culture has been shown to lead to deficits in cytoskeletal assembly, leading to arrested meiosis, increased levels of reactive oxygen species and cell death, and reduction in sperm binding [133]. In vitro exposure of mouse oocytes to PFHxS and PFOS at 600 μM has also been shown to induce deficits in meiosis due to alterations in spindle formation, elevating levels of reactive oxygen species, inducing chromosome misalignment, and compromising oocyte development [129]. Similarly, perfluorononanoic acid (PFNA) at 600 μM disrupted spindle assembly and indirectly induced mitochondrial oxidative stress, DNA damage, and apoptosis of oocytes [130]. Female mice given drinking water with 0.6 ng/L, 2.8 ng/L, or 4.4 ng/L of PFAS for 9 weeks exhibited diminished oocyte quality, embryogenesis, and a reduced number of blastocysts compared with the vehicle control [132]. In mice, PFOA exposure disrupted mitochondrial metabolism by reducing ATP levels, increasing ROS levels, and disrupting the mitochondrial membrane potential. In addition, the DNA damage marker γ-H2AX increased in oocytes of PFOA-exposed mice [130]. Mice administered a mixture of PFOA and PFOS at 0, 1 or 5 mg/kg/day for 21 days led to reduced time in estrus, smaller litter size and smaller body mass of resulting offspring [131]. This mixture also induced epigenetic alterations, increasing H3K9 and H3K27 acetylation levels, and increasing DNA damage in oocytes [131]. In addition, mitochondrial dysfunction led to oxidative stress and apoptosis in oocytes, likely contributing to the decrease in blastocyst quality and litter size [131]. Single-cell transcriptomic analysis revealed that this exposure also interrupted genes important for energy metabolism, cytoskeleton, apoptosis and the Hippo signaling pathway (a pathway which regulates apoptosis and oxidative stress) [131].

Collectively, PCBs and PFAS both disrupt core processes of oocyte maturation, including mitochondrial function, spindle assembly, epigenetic regulation, and oocyte-somatic cell communication, ultimately compromising developmental competence. AhR-dependent mechanisms likely contribute to the effects of dioxin-like PCBs (77, 126, 169) [120], while ROS may play a greater role in the effects of non-dioxin-like PCBs [122]. PFAS effects on oocytes appear to be more closely associated with mitochondrial dysfunction and oxidative stress, with less evidence for direct AhR involvement [128,129,130]. These molecular and cellular defects may translate into reduced fertilization rates, impaired embryogenesis, and diminished reproductive success. Consequently, chronic environmental exposure to these persistent contaminants likely exacerbates fertility challenges in both animals and humans.

## 4. Effects on Fertility, ART Outcomes and Pregnancy

While this review focuses on preclinical and mechanistic evidence for PCB and PFAS-induced reproductive toxicity, human data on fertility and ART outcomes are discussed where relevant to contextualize these findings. A comprehensive evaluation of human pregnancy outcomes falls outside the scope of this review. PCB exposure has been linked to reduced fertility and pregnancy complications. In an accidental exposure cohort, studies have found that women with higher PCB levels have fewer numbers of lifetime pregnancies; however, there were no associations with PCBs and pregnancy or birth outcomes [47]. Higher concentrations of PCB 28 and 180 in follicular fluid (FF) were associated with thinner endometrial thickness; PCB 28 was also associated with lower retrieval of oocytes, while PCB 180 was associated with reduced fertilization in intracytoplasmic sperm injection (ICSI) outcomes [134,135]. This study also found that PCB 52 in FF was negatively correlated with the number of implanted embryos [135]. Similarly, higher levels of some PCB congeners in FF of women were associated with reduced antral follicle count, estradiol concentration, endometrial thickness, number of oocytes retrieved, oocyte fertilization and embryo quality, embryo implantation and live birth [136,137]. Women undergoing in vitro fertilization for infertility with increased blood serum PCB levels had decreased estradiol (PCB 138, PCB 153 and PCB 180), decreased FSH (PCB 118 and PCB 138), and decreased LH (PCB 118, PCB 138, PCB 153 and PCB 180). Further, women without polycystic ovarian syndrome but unexplained reduced fertility had higher levels of PCB 118, PCB 153, PCB 138, PCB 180 versus those with explained factors of infertility [146]. Because rhesus macaques closely mirror human reproductive physiology and placental transfer mechanisms, they represent a highly translational model and have been used in reproductive toxicology studies, demonstrating that gestational PCB exposure is more detrimental than postnatal exposure. Accidental exposures to PCBs from concrete sealant caused abortions or stillbirths in rhesus macaques [147]. Offspring of suspected accidental PCB-poisoned rhesus monkey dams were weak and exhibited suppressed growth, with most dying soon after birth or several months later [147]. Aroclor 1248 fed to monkeys for two months at doses of 0, 2.5 and 5 ppm has been reported to alter length and cyclicity of the menstrual cycle and decrease conception rate in rhesus monkeys [139,142]. Aroclor 1254 was found to be associated with reduced conception rates and abortions in rhesus and cynomolgus monkeys [140,148]. Marmoset monkeys that were orally dosed with 0.1, 1, or 3 mg of 3,4,3′,4′-tetrachlorobiphenyl (PCB 77) showed an absence of corpora lutea [149]. In reproductive-aged rhesus macaques given 4 mg/kg/day of Clophen A30, ovulation was blocked in three out of the four PCB-treated animals and primary follicles were depleted [138]. There were no significant changes in serum P_4_, testosterone, LH, or FSH. However, some of the treated anovulatory animals did exhibit elevated levels of FSH in the later phase of the cycle with no P_4_ rise, which is characteristic of an absence of corpora lutea, and low E_2_ levels [138]. Female NHPs fed PCBs invariably have higher levels of urinary ketosteroids than controls [150], which is indicative of increased androgenic steroid hormone metabolism and thus may be associated with decreased serum E_2_ and/or P_4_ levels found in certain studies [151,152]. Furthermore, PCBs have been shown to interfere with fertility and pregnancy outcomes in cattle, mice, and rabbits [124,127,145]. Together, these data suggest that PCBs may influence fertility and pregnancy outcomes, but variations in doses and timing are important to consider in extrapolating findings across cohorts. Nonetheless, they raise important points about the effects of PCBs on health and fertility.

Exposure to PFAS negatively affects oocyte fertilization and leads to adverse pregnancy outcomes. PFAS accumulates in the body and interferes with the hormones and cellular processes essential for fertilization and pregnancy. Human exposure to PFOS has been shown to delay puberty, lead to oligomenorrhea, and longer time to pregnancy [153,154,155,156], which is also supported by animal studies [82,100]. Maternal concentrations of PFOA were negatively associated with oocyte yield, fertilization and embryo quality in women undergoing IVF; however, none of the PFAS were associated with probability of implantation, pregnancy or live birth [143,144]. Further, women undergoing IVF who had higher plasma PFNA and PFOS concentrations also had reduced number of retrieved oocytes and embryos, and PFHxS levels were associated with fewer pregnancies during embryo transfer [143]. PFAS were detected in FF from women undergoing IVF in Australia; PFOS and PFOA were detected in all samples at 4.9 and 2.4 ng/mL, respectively [157]. Interestingly, both positive and negative trends were observed for some infertility factors, dependent upon the type of PFAS [157]. In bovine cumulus–oocyte complexes (COCs) exposed to 0.01–100 µg/mL perfluorohexane sulfonate (PFHxS) in vitro, concentrations ≥ 40 µg/mL PFHxS decreased oocyte developmental competence, while concentrations at 1–10 µg/mL altered lipid distribution in blastocysts [125]. Genes affected by 0.1 µg/mL PFHxS included those involved in ROS generation, DNA methylation, and estrogenic and PPARγ pathways [125]. In vitro exposure of bovine COCs to 2 ng/g and 53 ng/g PFOS, corresponding to average and high human exposure respectively, led to delayed first cleavage to the two-cell stage and delayed blastocyst development [126]. In this exposure paradigm, blastocysts showed increased lipid volume after exposure to PFOS 53 ng/g but decreased average lipid droplet size with PFOS 2 ng/g [126]. Gene expression analyses from high PFOS identified dysregulated pathways related to cell death, stress response, differentiation and proliferation; DNA-methylation changes were associated with similar transcriptionally altered pathways, with one of the top pathways being tumor protein 53 (TP53) [126]. Notably, these effects may extend beyond the exposed generation, as maternal PCB and PFAS exposure has been associated with increased time to pregnancy in daughters and chromosomal abnormalities in offspring, suggesting intergenerational reproductive consequences [58,59].

The studies above indicate that PCBs and PFAS can adversely affect multiple aspects of female reproductive function, including impaired oocyte quality, fertilization, and ART outcomes across human and animal studies. Findings are not always consistent, particularly in human cohorts where several studies have reported no significant associations with live birth [47,143,144]. These differences may reflect variation in exposure levels and timing, species, study populations, and the complex nature of contemporary low-dose exposures compared with controlled experimental conditions. Congener and compound-specific effects further complicate direct comparison, as individual PCB and PFAS can produce opposing effects on similar reproductive endpoints [135,146,157]. Collectively, the evidence supports overlap in deleterious effects of PCBs and PFAS on female reproductive endpoints with likely shared and unique upstream pathways leading to the observed effects.

## 5. Comparing Experimental Exposures to Regulatory Guidelines

It is important to consider the relevance of experimental findings to human health in a regulatory context. The Joint FAO/WHO has set a tolerable monthly intake of 70 pg/kg for dioxin-like PCBs [158]. The U.S. Environmental Protection Agency has fixed maximum contaminant levels for PFAS in drinking water with 4 parts per trillion for PFOS and PFOA, and 10 parts per trillion for PFNA, PFHxS, and HFPO-DA [159]. When these regulatory standards are compared with many of the mechanistic studies discussed above, a substantial gap becomes apparent. Several in vitro studies describe the effects of PFAS concentrations in the hundreds of micromolar ranges, which can be orders of magnitude higher than concentrations typically detected in human serum [128,129,130]. In contrast, studies reflecting PFOS concentrations of average (2 ng/g) and high (53 ng/g) human exposure demonstrated visible effects on bovine oocyte development, suggesting that some reproductive outcomes are sensitive to PFAS at low levels [19]. Differences in exposure levels should be considered when interpreting experimental findings. Findings obtained at supraphysiologic concentrations are important for identifying candidate pathways of toxicity, but they should not be assumed to predict effects at lower exposure levels. Future studies using exposure concentrations that display current biomonitoring data, with continued improvement of regulatory thresholds as new mechanistic evidence accumulates, will be needed to close this gap between laboratory findings and population-level risk assessment.

## 6. Conclusions and Future Perspectives

Despite production bans, PCBs and PFAS have remained prevalent in our society and have effects on female reproduction that are still notable today. Because of their chemical stability, both groups persist in the environment and in the body for many years. The studies reviewed here provide evidence that PCBs and PFAS are capable of interfering with steroid hormone production in granulosa, theca, and luteal cells; can change levels of estradiol, progesterone, and androgens; and act on hormone receptors such as ER, AR, AhR, and PPARs. These molecular changes likely disturb follicle growth, reduce the ovarian reserve, and damage oocyte structure and function. Another important concern is that both chemical classes can pass to the next generation during pregnancy and lactation, causing changes in hormone levels, ovarian function, and even DNA methylation and gene expression in offspring, with consequences for their fertility and menstrual cycles later in life. Despite this growing evidence, important gaps remain. Many studies focus on single congeners or single PFAS, whereas humans are exposed to complex mixtures. Data on low-dose, long-term exposure, combined PCB and PFAS effects, and diverse human populations are still limited. More work is needed to connect mechanistic findings from animal, NHP and in vitro models with real-world exposure scenarios and clinical outcomes, including subtle changes in cycle regularity, ovarian reserve, and success in pregnancy.

## Figures and Tables

**Table 1:** Effects of PCBs and PFAS on reproductive abnormalities in mammals.

PCBs/PFAS	Species	Effects		
		Folliculogenesis	Oocyte Maturation	Fertilization
Aroclor-1254, Aroclor 1248, PCB 77, PCB 126, PCB 153	Bovine		↓ oocyte maturation [114,118] ↑ cumulus cells apoptosis [120]	↓ fertilization, cleaved, blastocyst [114,118,124] ↑ polyspermy [114] ↓ implantation [124]
PFOS, PFHxS	Bovine			↓ embryo, blastocyst [125,126]
Aroclor-1254, Aroclor-1221, Aroclor-1268, PCB 118	Mice		↑ spindle length, cumulus cell DNA damage [116] ↑ cumulus cell apoptosis [117]	↓ fertilization, embryo growth [127]
PFDA, PFHxS, PFOS, PFNA, PFAS, PFOA, PFBS	Mice	↓ mature follicles, corpora luteum [82,105,106] ↑ atretic follicles [103]	↓ maturation-promoting factors, polar body extrusion, spindle assembly checkpoint, cytoskeleton, mitochondrial function [128,129,130,131] ↑ ROS, DNA damage, oocyte apoptosis [128,132]	↓ blastocyst, litter size [132]
Aroclor-1254, PCB 126, PCB 153	Swine	↓ estradiol secretion, follicular development [83]	↓ cytoplasmic network, communication between oocytes and granulosa cells [119]	↓ blastocyst [119]
PFOS	Swine		↓ cytoskeleton, mitochondrial function, meiotic competency [133] ↑ ROS, apoptosis [133]	↓ fertilization [133]
PCB 28, PCB 180, PCB 52, PCB 64, PCB 77, PCB 101, PCB 146, PCB 47, PCB 158, PCB 153, PCB 187, PCB 156, PCB 99, PCB 105, PCB 138, PCB 183, PCBs 74, PCB 99 and PCB 187	Human	↓ non-growing follicle [134]		↓ fertilization, embryo quality, embryo implantation, pregnancy [135,136,137]
Clophen A 30, Aroclor 1248, Aroclor 1254	Macaques	Block ovulation [138,139]		↓ conception rate, pregnancy rate [140,141] ↑ abortions or stillbirths [140,142]
PFOA, PFNA, PFOS, PFHxS, PFUnA, EtFOSAA	Human		↓ mature oocytes [143,144]	↓ fertilization, embryo, pregnancy [143]
A1221, Aroclor 1016, PCB 126	Rat	↓ primordial, secondary, antral follicle and corpora lutea number [78,97,98]		
PFOA	Rat	↓ primordial follicles, growing follicles and corpora lutea [104]		
Aroclor 1260	Rabbit			↓ embryonic development [145]

Perfluorodecanoic acid (PFDA); perfluorohexanesulfonic acid (PFHxS); perfluorooctanesulfonic acid (PFOS); perfluorononanoic acid (PFNA); perfluorooctanoic acid (PFOA); per- and polyfluoroalkyl substances (PFAS); perfluoroundecanoic acid (PFUnA); N-ethylperfluorooctanesulfonamidoacetic acid (EtFOSAA); perfluorobutanesulfonic acid (PFBS). ↑ indicates increase, ↓ indicates decrease.

## Data Availability

No new data were created or analyzed in this study. Data sharing is not applicable to this article.
